# Novel Microemulsions with Essential Oils for Environmentally Friendly Cleaning of Copper Cultural Heritage Artifacts

**DOI:** 10.3390/nano13172430

**Published:** 2023-08-26

**Authors:** Mihaela Ioan, Dan Florin Anghel, Ioana Catalina Gifu, Elvira Alexandrescu, Cristian Petcu, Lia Mara Diţu, Georgiana Alexandra Sanda, Daniela Bala, Ludmila Otilia Cinteza

**Affiliations:** 1 “Ilie Murgulescu” Institute of Physical Chemistry, Romanian Academy, 202 Spl. Independentei, 060021 Bucharest, Romania; mihaela4ioan@yahoo.com (M.I.); dan.florin.anghel@gmail.com (D.F.A.); 2 Polymer Department, National Institute for Research and Development in Chemistry and Petrochemistry—ICECHIM, 202 Spl. Independentei, 060021 Bucharest, Romania; catalina.gifu@icechim-pd.ro (I.C.G.); elvira.alexandrescu@icechim.ro (E.A.); 3 Microbiology Department, Faculty of Biology, University of Bucharest, 030018 Bucharest, Romania; lia-mara.ditu@bio.unibuc.ro; 4 Physical Chemistry Department, University of Bucharest, 4–12 Blv. Regina Elisabeta, 030018 Bucharest, Romania; georgiana-alexandra.sanda@s.unibuc.ro (G.A.S.); dbala@gw-chimie.math.unibuc.ro (D.B.)

**Keywords:** microemulsion, metallic cultural heritage artifacts, essential oils, antifungal, anticorrosive

## Abstract

Cleaning represents an important and challenging operation in the conservation of cultural heritage, and at present, a key issue consists in the development of more sustainable, “green” materials and methods to perform it. In the present work, a novel xylene-in-water microemulsion based on nonionic surfactants with low toxicity was obtained, designed as low-impact cleaning agent for metallic historic objects. Phase diagram of the mixtures containing polyoxyethylene-polyoxypropilene triblock copolymer Pluronic P84 and D-alpha-tocopheryl polyethylene glycol 1000 succinate (TPGS) as surfactants, water, ethanol and xylene was studied, and a microemulsion with low surfactant content was selected as suitable cleaning nanosystem. Essential oils (EOs) from thyme and cinnamon leaf were added to the selected microemulsion in order to include other beneficial properties such as anticorrosive and antifungal protection. The microemulsions with or without EOs were characterized by size, size distribution and zeta potential. The cleaning efficacy of the tested microemulsions was assessed based on their ability to remove two types of artificial dirt by using X-ray energy dispersion spectrometry (EDX), scanning electron microscopy (SEM), contact angle measurements and color analysis. Microemulsions exhibit high capacity to remove artificial dirt from model copper coupons in spite of very low content of the organic solvent. Both thyme and cinnamon oil loading microemulsions prove to significantly reduce the corrosion rate of treated metallic plates compared to those of bare copper. The antifungal activity of the novel type of microemulsion was evaluated against *Aspergillus niger*, reported as main treat in biocorrosion of historic copper artifacts. Application of microemulsion with small amounts of EOs on Cu plates inhibits the growth of fungi, providing a good fungicidal effect.

## 1. Introduction

Contrary to popular opinion, metals are among the sensitive materials from which cultural heritage objects can be made, as they are currently subjected to various corrosion processes and to the development of fungi and other biological pests. Historic metallic artifacts are also affected by the presence of adherent and non-adherent deposits (dust, smoke, wax, etc.) which, in addition to physical and chemical damage, lead to significant unaesthetic changes in the original appearance of the objects [1].

Cleaning is considered one of the most delicate and challenging steps in the conservation and restoration of cultural heritage. Thus, the selection of the most adequate cleaning method depends on many factors, such as the object’s materials and its state of degradation, other characteristics of the object (the size, the surface topography) and the nature of the dirt to be removed [2]. A particular situation is encountered in the case of sacred art heritage objects, hosted by churches in use, not in museums. They are very often exposed to the accumulation of dirt, consisting of a mixture of dust deposits, carbon residues and various greasy materials, especially wax and oils, produced by burned candles.

A cleaning procedure usually recommended to remove this kind of oily dirt is based on the employment of an organic solvent in various ways such as soaking, poultice or cotton swab applications. Common solvents dimethyl formamide and petroleum spirits are listed as red (ranking score 1) while xylenes are listed as orange (ranking score 2) in the “green” metrics list provided by industrial and academic institutions [3,4], far from the desired ranking as green solvents.

Since the cleaning of artistic or historic objects is a meticulous, long-term procedure, concerns on the health risks on the conservation practitioners have been expressed in the recent decades, leading to extensive research devoted to the development of safer solutions, with less toxic chemicals, reduced volumes and low impact on the environment [5].

One of the most popular approaches to reduce the amount of toxic organic solvents is the use of microemulsions as nanostructured cleaning solutions. Oil-in-water microemulsions such as L/L colloidal systems with oily droplet sizes of less than 100 nm dispersed in aqueous media exhibit numerous advantages as cleaning agents due to their easiness in preparation, high efficiency in grime/soil removal and low content of organic solvent. Various O/W microemulsions were studied by Baglioni et al. [6,7,8,9], and remarkable results were reported in the removal of aged polymeric consolidants from wall paintings. W/O microemulsions were prepared and successfully tested as cleaning agents for contemporary acrylic-based paintings by Ormsby et al. [10]. O/W microemulsions proposed by Baglioni et al. usually contain organic solvents, such as *p*-xylene, ethyl acetate or commercial mixtures of solvents in concentrations of less than 10%, while various surfactants and cosurfactants in different concentrations are used. In spite of the significant advantages proved by the employment of microemulsion for cleaning painted surfaces, there are some disadvantages of these nanosystems related to the high content of water spread to the work of the art substrate and the retention of small quantities of surfactants. To overcome this limitation, microemulsions were encapsulated in hydrogels or in halloysite particles embedded in polymer membranes [11].

The potential alteration that could be caused by an artwork being exposed to either aqueous-based or non-polar solvents has generated a particular interest in the case of painted surfaces (such as frescoes or paintings). As a result, a significant body of research has been conducted on this topic [12,13], whereas the interaction between cleaning solvents and metal objects has received significantly less attention.

The major threat to the preservation of historic metal artifacts are corrosion processes triggered by various common conditions, usually the humid environment, which are frequently investigated [14]. Considerably fewer studies have been published on the corrosion caused by greasy deposits, such as grime mixed with oily residues or by the microbial colonization of the metal surface.

Soot and smoke which are produced during the incomplete combustion of various materials (such as candles) contain acids, carbonaceous chemicals, dust and grease. The presence of soot in the soil where archaeological items are discovered was found to be responsible for the increase in the soil acidity, and, consequently the increased corrosion process of metallic objects [15,16].

Biological attacks occur in closed, dark spaces with high humidity, and generally develop on stone or organic material surfaces, generating huge damages in the integrity of cultural heritage items [17]. However, in some cases, fungi such as *Aspergillus niger* have been also discovered on the surfaces of metal objects such as aluminum [18,19], copper [20], zinc [21], or steel [22]. The acids produced by the fungi lead to biocorrosion processes, expressed as solubilization of metal, copper in particular [20]. Additionally, the presence of fungal colonies disturbs the visual appearance of the artistic objects and produces respiratory diseases, endangering the people who handle them [23].

A novel trend regarding the sustainable, “green” approach in conservation of cultural heritage involves ecological treatments based on natural products which have already proven anticorrosive and antimicrobial efficiency in other fields. A large variety of essential oils [24], such as oils from oregano [25], thyme [26], cinnamon, pomegranate [27], curcuma [28], sage [29], and clove [30] were intensively studied. Many of them are used as commercial products in the form of antimicrobial pharmaceuticals or biocides in industrial applications. Among them, thyme and cinnamon essential oils have been reported as natural products with exceptional antibacterial and antifungal activity against many microorganisms. Some of essential oils, including thyme and cinnamon oils, display antifungal or fungicidal activity against various strains of *Aspergillus* [31]. Additionally, the latter was recently reported to act as eco-friendly anticorrosion treatment for mild steel, with good performance as protective coating [32].

In this work, a novel microemulsion was prepared as a solution to replace the commonly used organic solvent mixtures for cleaning grime and oily deposits from the metallic artistic or archaeological artifacts. The nanosystems contain a very-low-oil phase and a moderate amount of nontoxic surfactants, while essential oils from thyme or cinnamon are encapsulated as additional oils, acting also as antifungal and anticorrosive agents. To the best of our knowledge, there is no paper in the literature reporting the use of microemulsions in the cleaning of metallic historic artefacts.

The novel multifunctional microemulsion was evaluated in terms of cleaning efficiency and impact on the properties of copper samples as model metal, with particular interest in the protective effect against corrosion and fungal colonization.

## 2. Materials and Methods

### 2.1. Reagents

Polyoxyethylene-polyoxypropilene triblock copolymer (PEO–PPO–PEO) Pluronic P84 and *p*-xylene (purity > 99.5%) were purchased from Sigma Aldrich (Merck Group, Darmstadt, Germany). D-alpha-tocopheryl polyethylene glycol 1000 succinate (TPGS) was kindly gifted by Antares Health Products, Inc. (Frankfurt am Main, Germany). Ethanol was purchased from ChimReactiv S.R.L. (Bucharest, Romania). Essential oils of thyme and cinnamon were purchased from Ellemental (Oradea, Romania). Incralac laquer (Incralac 44) was purchased from CTS Europe (Florance, Italy).

All reagents were used as received, without further purification.

Distilled water was obtained using a Milli-Q Advantage A10 system (Merck Millipore, Darmstadt, Germany).

Copper foils (copper 99.99% purity) purchased from Tianjin Blueprints Iron and Steel Co., Ltd. (Tianjin, China) were used as the metallic material for artificial dirt deposition and cleaning procedure.

### 2.2. Microemulsion Preparation

For the preparation of the microemulsions, a mixture of two polymeric surfactant Pluronic P84 and TPGS was used in a 1:1.2 molar ratio. The chemical formula and some of their physico-chemical characteristics are listed in Table 1.

The microemulsions were prepared in two steps. First, the appropriate amount of surfactants (Pluronic P84 and TPGS) was mixed in water, at room temperature, under vigorous magnetic stirring that lasted 24 h in order to obtain a clear, homogeneous micellar dispersion. Then, to these systems, *p*-xylene was added as an oily phase to form the desired microemulsions.

The phase diagram of the W-Xy-P84-TPGS system was built by recording the systems prepared when diluting the original sample of surfactant/cosurfactant with oil. Several dilution lines were investigated starting with surfactant solutions with the same molar ratio (P84-TPGS 1:1.2) with concentrations ranging from 10 to 40%. The identification of systems as microemulsions, emulsions or viscous phases (gels or lyotropic liquid crystals) was performed by visual observation, phase separation and viscosity.

To prepare essential oil encapsulating microemulsions, different volumes of commercial essential oil (EO) were further added to the prepared microemulsions until the maximum solubilization was achieved. Samples of oil swollen polymeric micelles were prepared by adding small amount of essential oils in the micellar dispersions under magnetic stirring until isotropic systems were obtained.

### 2.3. Cleaning Procedure

The cleaning efficiency was tested on copper coupons as model metallic samples. The Cu coupons were cut from the as-purchased foils in 20 × 50 mm rectangles for cleaning tests, and in 3.5 × 50 mm rectangles for further electrochemical measurements. The copper samples were washed using commercial detergents, polished with sandpaper (Klingspor LS 309 XH, from Klingspor AG, Haiger, Germany), and finally degreased and rinsed with acetone and ethanol.

The artificial dirt used to ensure the contamination of the metal samples in the laboratory was prepared to mimic the composition of layered dirt deposited on metallic artifacts in ecclesiastical spaces (churches, monasteries) or deposited on the outdoor monuments located in polluted areas. The recipe was adapted from the studies published by Wolbers [34] and Ormsby [35]. It consists of a mixture of 1% guar gum, 4% paraffin oil, 2% carbon black and 2% bee wax dispersed in iso-octane. The artificial dirt with carbon black content is designed to mimic the composition of dirt contaminating objects in spaces where grime is present, and it is denoted as MSA, while another artificial dirt without carbon black, denoted as MSC, mimics the oily layered dirt deposited on some artistic objects during the storage and handling.

The prepared artificial dirt was deposited on the copper samples by brushing. Five successive layers were applied in order to obtain medium–heavy contaminated surfaces as homogeneously as possible. The dirt coated copper coupons were let to dry 24 h before the cleaning test were performed.

The procedure of the removal of the deposited artificial dirt required cotton swabs soaked in the microemulsions as cleaning solutions, which is considered to be the closest to the real method used in conservation/restoration practice [10]. The cotton swabs were rolled over the surface of the Cu pieces for approximately 10 s with light pressure. The cleaning procedure was repeated five times on the same area, using different cotton swabs each time. No rinse step was performed in order to evaluate the retention of the cleaning microemulsion. Finally, a dry cotton swab was rolled on the metal surface in order to remove the excess of microemulsion from the copper samples.

### 2.4. Characterization

DLS: Pure and EO loaded microemulsions were evaluated for their particle size, polydispersity index and zeta potential using the dynamic light scattering (DLS) and electrophoretic light scattering methods. Experiments were performed at 37 °C without any further dilution of the samples. All measurements were performed using a Nano ZS Zetasizer (Malvern Instruments Ltd., Malvern, UK) equipped with a He-Ne laser of 532 nm at a scattering angle of 173°. Folded capillary cells were used for zeta potential measurements, while quartz standard cuvettes were used for size measurements.

Contact angle: The hydrophobicity of the surfaces was assessed using contact angle values, with water as a reference liquid. The measurements were performed on contact angle instrument OCA 15 (DataPhysics, Filderstadt, Germany). The static contact angles of water on deposited films were obtained using the sessile drop method at room temperature. The reported contact angle values were obtained by analyzing the captured images, fitted with suitable equations provided by the instrument software (SCA 202 version 3.11.6). The reported contact angle main values were computed as the average of five measurements (liquid droplets placed in various regions of the film surface).

SEM and EDX: Scanning electron microscopy (SEM) was used to evaluate the morphology of the samples and the surface analysis. The images were captured on an FEI Quanta 200 instrument (Eindhoven, The Netherlands) at a 30 kV accelerating voltage, using a large field detector (LFD) and a low-vacuum working mode. X-ray energy dispersion spectrometry was performed using an instrument consisting of a field-emission scanning electron microscope, Hitachi TM4000plus II (Hitachi, Hokkaido, Japan), coupled to a spectrometer by energy-dispersive X-ray (EDX). A backscattered electron (BSE) detector was used at an accelerating voltage of 15 kV, at a magnification of 200.

UV-Vis spectra were recorded on a JASCO V-570 spectrophotometer (Jasco, Easton, MD, USA) in a range of 380 to 780 nm using Spectralon as a standard. Colorimetric parameters (in the CIELab color space) were calculated using the software of the instrument (Spectra Manager version 1.02.05).

Electrochemical study: Electrochemical measurements were performed using a potentiostat-galvanostat system Autolab PGStat 12 controlled by the General Purpose Electrochemical System (GPES) with Windows interface (version 4.9.007). Potentiodynamic polarization curves were plotted from −0.8 to 0.4 V/Ag/AgCl at a polarization scan rate of 10 mV/second. Three electrodes in a one-compartment cell (10 mL) were used in all experiments: the reference electrode was Ag/AgCl, the counter electrode was platinum with a large area and working electrodes were bare and coated copper plates. All potential values described in this study were referred to an Ag/AgCl electrode. The experiments were performed without stirring at 25 ± 2 °C. Each electrochemical experiment was carried out in triplicate and the determined parameters represent the average of the measurements.

Corrosion process was simulated in a saline medium (3.5% NaCl solution) at room temperature. The copper plates used as working electrodes were uncoated (Cu) and coated with various EO-loaded nanosystems (mixed micelles and micoremulsions) or with EOs as references. Before each test, the exposed surface of copper plates was polished using SiC paper (from grade 240 to grade 1000), rinsed with distilled water, ultrasonicated in acetone and dried at room temperature. The cleaned copper plates were covered with two layers of each type of coating and left to dry for two weeks. Linear sweep voltammetry (LSV) was used to evaluate the corrosion process by sweeping the potential applied at a working electrode (Cu without and with several types of coatings) and measuring the current response. Electrochemical parameters were obtained using instrument software (Autolab Software version 4.9) and used for evaluating the susceptibility of metallic support based on copper to corrosion.

Antifungal activity: Antimicrobial activity of the microemulsions and associated nanosystems was evaluated against *Aspergillus niger*, selected because it is the most common microorganism colonizing copper historic artifacts. Filamentous fungal strain was represented by the opportunistic species *Aspergillus niger* 103 isolated from environment and included in the Microbial collection of Microbiology department, Faculty of Biology, University of Bucharest.

In order to carry out the screening of the antifungal activity of the obtained samples, filamentous fungi spore suspensions were prepared from fresh cultures of 5–7 days developed in a solid PDA medium (Potato Dextrose Agar suitable for microbiology, NutriSelect^®^ Plus, Merck). To obtain the spore suspensions, 10 mL of sterile physiological water (SPW) with 1 drop of Tween 20 (10 µL) was added over the 5–7-day fungal culture, followed by shaking to detach the spores. Afterwards, the obtained dense spore suspensions were recovered in another sterile tube, kept at rest for 1 min to decant the fungal filaments; from the supernatants represented by the spore suspension, 1:50 (*v*:*v*) dilutions were made in SPW. The density of the spore suspensions was adjusted to an optical density (at 620 nm) between 0.09 and 0.11 (corresponding to the McFarland turbidity standard 0.5, using DEN-1B McFarland densitometer) in order to reach spore density between 0.4 × 10^4^ UFC/mL and 5 × 10^4^ UFC/mL (according to the NCCLS Reference Method for Broth Dilution Antifungal Susceptibility testing of Filamentous Fungi) [36].

Afterwards, the antifungal activity was tested using an adapted diffusion method, which involves the spread of the fungal suspensions on the surface of the Sabouraud solid medium (Scharlau Microbiology) and placing it over 10 μL of each tested sample. The plates were incubated for 5–7 days at room temperature and checked daily to observe the growth of the fungal mycelium.

The qualitative evaluation of the antifungal activity of the tested samples was carried out at different time intervals after incubation: 48 h, 72 h and 5 days, both by macroscopic observation of fungal culture and by microscopic analysis of the fungal mycelium, using an inverted microscope (Zeiss AXIO inverted microscope, with 100× magnification power), followed by the determination of the inhibition zone diameters (expressed in mm). For the inhibition zones appearing around the spot of the tested sample, the diameters were determined and expressed in mm.

The quantitative method for testing antifungal activity by establishing minimum inhibitory concentration (MIC) values was carried out according to the recommendations of the standard NCCLS Reference Method for Broth Dilution Antifungal Susceptibility testing of Filamentous Fungi [36].

To determine the CMI value, binary dilutions of the test compounds/suspensions/solutions were conducted in an RPMI 1640 medium (RPMI-1640 Medium, With L-glutamine and sodium bicarbonate, liquid, sterile-filtered, Sigma-Aldrich, St. Louis, MI, USA). Each well was subsequently inoculated with fungal suspension at standard density, established according to the procedure described in the previous step, respecting the volumetric ratio 10/1 = RPMI/spore suspension. The samples were incubated for 5–7 days at room temperature.

In parallel, following the same steps and the same reaction conditions, two control samples were tested: microbial growth control (MC) (wells containing exclusively culture medium inoculated with spore suspension) and medium sterility control (MS) (wells containing exclusively culture medium).

The minimum inhibitory concentration was established as the last concentration at which no fungal growth was observed, i.e., the appearance of turbidity of the medium, accompanied or not by the color change from cyclamen to yellow (for *Aspergillus niger* strain). The absence of fungal hyphae growth was also microscopically confirmed via analysis of the wells with inverted microscope Zeiss AXIO (Carl Zeiss GmbH, Oberkochen, Germany) with a 250× magnification.

Statistical analysis was performed by using one-way analysis of variance (ANOVA) and *t*-test using the Data Analysis module in Excel. A *p*-value < 0.05 was considered statistically significant.

## 3. Results and Discussion

### 3.1. Phase Diagram of W-Xylene-P84-TPGS System

In this work, the obtention of single-phase microemulsions was targeted using the combination of Pluronic P84 and TPGS as a surfactant/cosurfactant mixture. Xylene was chosen as an oily phase since it is the organic solvent most used in the cleaning procedure applied to metallic artifacts.

Microemulsion formation was already reported in the systems water–Pluronic P84-xylene at surfactant concentrations up to approximately 35%, but with very a small amount of the oily phase [37]. In order to reduce the surfactant content needed to produce isotropic, thermodynamic stable systems, microemulsions, and to increase the oil content, TPGS was added as a cosurfactant.

**Figure 1:**
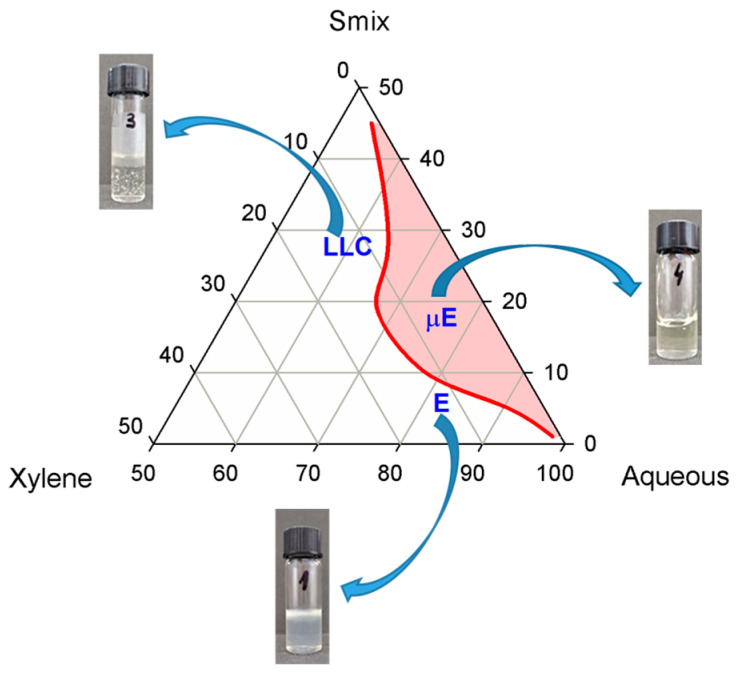
Phase diagram of water–xylene–Pluronic P84-TPGS (surfactant–cosurfactant molar ratio 1:1.2) system at 25 °C.

The phase diagram of the W-Xy-P84-TPGS system was built by diluting with xylene the aqueous solutions of P84-TPGS with a molar ratio of 1:1.2. The surfactant–cosurfactant molar ratio was selected from our previous screening experiments as suitable in terms of replacement of more toxic Pluronic surfactant with TPGS, a less harmful one, at the same time allowing the formation of a microemulsion. A reduced phase diagram of the system containing water–xylene–Pluronic P84-TPGS is shown in Figure 1.

The microemulsion domain is expanded compared with that of the phase diagram of water–xylene–Pluronic P84 [37]. The visual inspection of the fluid systems showed no evidence of phase separation; thus, Winsor IV (single-phase) microemulsions were obtained. Electrical conductivity measurements performed on a Cole-Parmer 500 conductivity meter (Cole-Parmer Instrument Co. Europe, St. Neots, UK) provided high values for all fluid samples, in the range of 4.37–5.84 mS/cm, suggesting that these are O/W-type microemulsions.

From the phase diagram, it can be seen that in the case of the P84-TPGS mixture, the content of the oily phase in the microemulsion region was rather reduced, up to 5.5%, similar to the system containing only Pluronic as a surfactant. At low surfactant–cosurfactant content, the increase in xylene concentration, ranging from 6 to 7.7% lead to the formation of emulsions.

The phase diagram was built in the region of surfactant mixture concentrations up to 50% since at high surfactant concentration only very viscous systems were obtained. Lyotropic liquid crystals (LLC), both hexagonal and lamellar type, were reported for systems containing Pluronic P84 at concentrations ranging from 44 to 87% [37]. In the case of our W-Xy-P84-TPGS pseudo-ternary system, at concentrations beyond 15% of the P84-TPGS mixture, LLC phases were present, accommodating the low amount of xylene (5–6%). Polarized optical microscopy images recorded on these samples revealed specific patterns for anisotropic liquid crystalline mesophases. No further investigations were performed on these systems, because the purpose of this study was to develop microemulsions as nanostructured cleaning solutions to be used in conservation practice.

Because one of the goals of the study was to reduce the oil content in the cleaning solution, the region of the phase diagram rich in the oily phase was not investigated, since increasing the oil ratio in water–xylene–Pluronic systems favors the formation of W/O type microemulsions, which cannot be used as a replacement of organic solvent systems.

The selection of the microemulsion sample subjected to further studies was performed based on the requirements for an ecological, cost-effective and less toxic cleaning system: (i) an adequate oil content (enough to ensure cleaning efficiency, but not so high to produce harmful effects on personnel performing cleaning procedures), (ii) low viscosity (in order to facilitate spreading), (iii) lowest content of surfactant–cosurfactant mixture (in order to minimize the environmental impact and to reduce the product’s price).

For the cleaning procedure applied to oil paintings or other sensitive textile or paper materials from cultural heritage objects, the use of more viscous cleaning agents is considered more adequate [38]. On the contrary, in order to ensure a rapid and efficient cleaning of metallic artifacts, a fluid cleaning agent could be more convenient; thus, the microemulsion nanosystem was selected.

### 3.2. Preparation of Microemulsions with Essential Oils

In order to obtain a multifunctional microemulsion, essential oils were tested as an added ingredient to provide more natural oily components to the cleaning function, together with the anticorrosive and antifungal properties of the cleaning nanosystem.

Due to their complex composition and the presence of large molecules with numerous functional groups, the essential oils are known to be very difficult to solubilize in diluted surfactant solutions. The maximum solubility of EOs in microemulsions was tested by adding, successively, volumes of the two essential oils until the original microemulsion turned into a slightly opalescent nanoemulsion. The results were similar for the two essential oils, with the maximum solubility of the thyme essential oil in the selected microemulsion containing a 11.35% surfactant mixture (P84-TPGS in 1:1.2 molar ratio) and the content of 4.5% xylene was 11.9 ± 0.6 mg/mL, while for the cinnamon essential oil, the value was found to be 13.3 ± 1.6 mg/mL.

The microemulsions with xylene and essential oil concentrations close to the maximum solubility were selected for further experiment, and their composition is listed in Table 2.

### 3.3. Characterization of the Selected Microemulsions

The selected microemulsions (only with xylene or also containing encapsulated essential oils) were characterized in terms of size, size distribution and droplet surface potentials compared to micelles from the same mixture of surfactants with EOs. The results are presented in Table 3.

**Figure 2:**
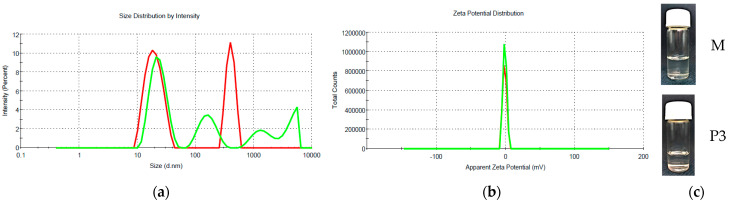
Size, size distribution (**a**); zeta potential (**b**) of xylene containing microemulsion (sample M—red lines) and microemulsion encapsulating thyme essential oil (sample P3—green lines); (**c**) visual aspect of microemulsions.

In Figure 2, the variations of the size, zeta potential and visual aspect of the representative microemulsion with EO from thyme are presented.

In the DLS diagrams in the intensity mode recorded for microemulsions, bimodal or multimodal distributions are observed as can be seen in Figure 2a for the microemulsion with thyme essential oil. The first signal is situated in the region of 19–42 nm assigned to the oily droplets, and one or two signals are found at larger values (hundreds of nanometers), probably due to the presence of aggregates/clusters of droplets. The extent of this second population was checked on the DLS diagrams in the number mode, when all these signals at high dimensions disappear (Figure S1 in Supplementary Materials), suggesting that the system contains, in the majority, oily droplets, and only a negligible proportion of some aggregates.

The O/W microemulsion containing only xylene (sample M) showed oily droplets with an average size of 19.16 ± 0.88 nm, which is consistent with other results in the literature [39,40]. The encapsulation of the essential oils in the xylene microemulsion lead to a slightly, but statistically significant increase (*p* < 0.05) in the main size for thyme oil (23.08 ± 0.5 nm), while in the case of cinnamon oil, a more pronounced increase was observed (42.89 ± 2.59 nm).

The nanosystems prepared as P84-TPGS mixed micelles encapsulating the same amount on EOs as microemulsions showed significantly smaller size for the surfactant aggregates, 12.65 ± 1.09 nm and 11.24 ± 1.75 nm. These values confirm the presence of the micelles with solubilized oil.

The prepared microemulsions show no signs of changes in the visual aspect and in the average size of the oil droplets during refrigerated storage for 4 weeks.

The zeta potential of the xylene-based microemulsion was found to be very low, −1.18 ± 0.03 mV, as is expected for a system stabilized with nonionic surfactants Pluronic P84 and TPGS. The small value of surface potential could explain the presence of some aggregates detected in the DLS diagram, as it is reported for other microemulsion and micellar systems with Pluronic derivatives [41].

The EO encapsulating microemulsions show similar zeta potential values, suggesting that the essential oils are entrapped into the oily droplets of the microemulsions.

### 3.4. Cleaning Efficiency Assessment

The prepared microemulsions were tested as cleaning agents on the copper samples covered with two types of artificial dirt, namely MSC and MSA, in order to mimic greasy deposited layers and grime-mimicking dirt, respectively.

**Figure 3:**
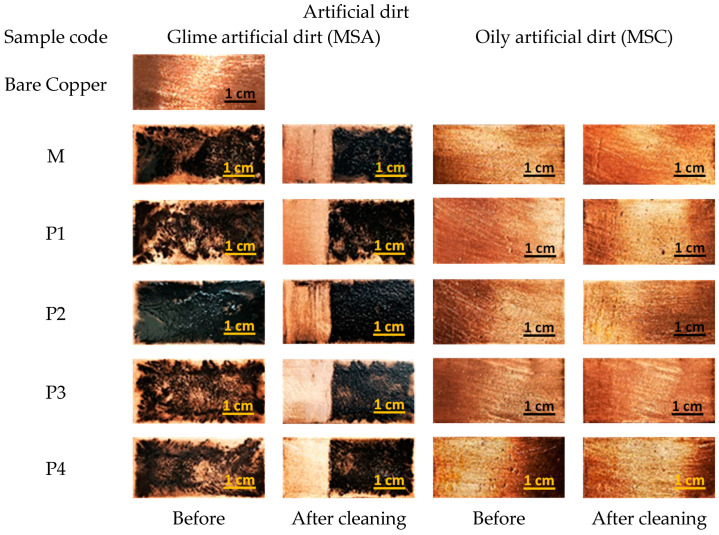
Visual aspect of Cu samples contaminated with various types of artificial dirt, before and after cleaning with microemulsions. In the third and fifth columns, the left part of the Cu sample was subjected to cleaning procedure, while the right part was left for comparison.

In Figure 3, the visual aspect of the metal samples is shown before and after the cleaning treatment. The cleaning procedure was performed as described earlier, with cotton swabs soaked in various nanosystems containing xylene and essential oils.

Upon visual inspection, it can be observeed that all surfactant-based nanosystems, either P84-TPGS mixed micelles with essential oil solubilized or xylene P84-TPGS microemulsions, pure or with EOs, show good capacity for cleaning grime artificial dirt, even at a low number of sequences (five times). Regarding the aspect of copper plates contaminated with oily artificial dirt, the cleaning results are difficult to observe due to the shiny aspect and the color of the metal surface.

As a preliminary evaluation of the cleaning effect, the morphology and the composition of the surface material deposited on the copper coupons were investigated by using SEM and EDX analyses.

**Figure 4:**
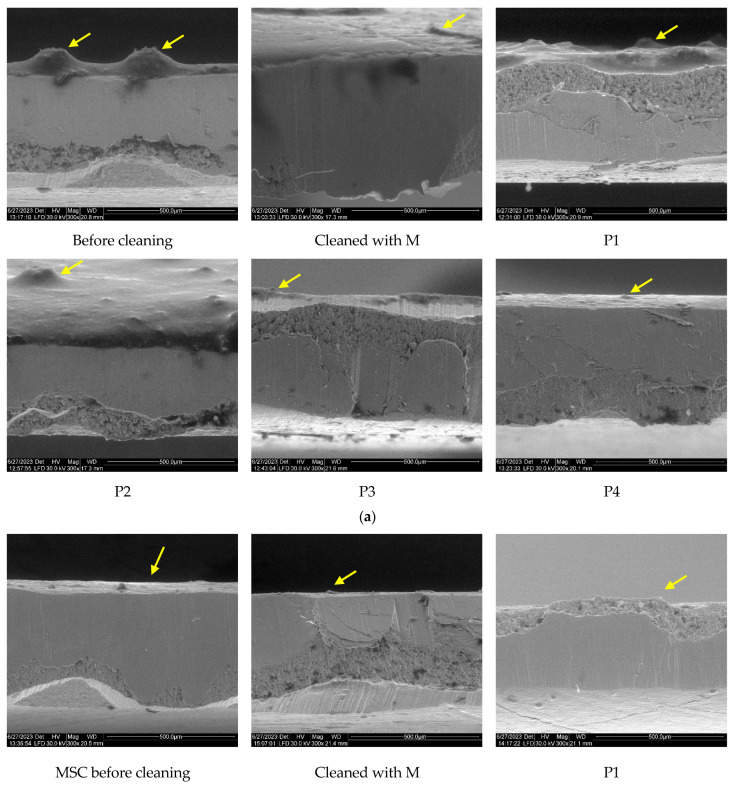

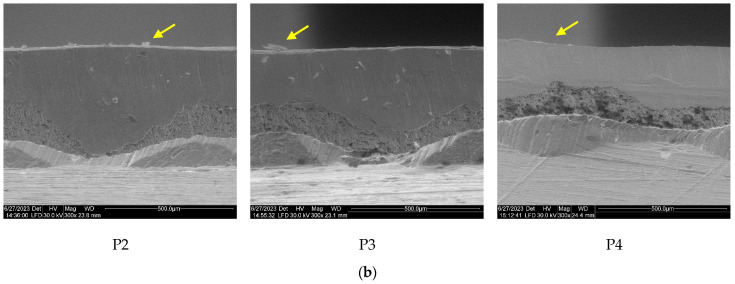
(**a**) SEM images of cleaned and dirt exposed Cu samples using grime-like artificial dirt (MSA). (**b**) SEM images of cleaned and dirt exposed Cu samples using oily artificial dirt (MSC). Yellow arrows indicated the residual dirt.

In Figure 4a,b, the cross-section of samples contaminated with artificial dirt is shown compared to the region subjected to the cleaning procedure.

In the SEM images of the cross-section of copper coupons covered with MSA artificial dirt (Figure 4a), a consistent layer with a specific morphology (rough layer with carbon particles embedded) can be observed. In images of the plates subjected to the cleaning procedure, a significant reduction in the dirt layer is to be noted, with no obvious differences when using microemulsions with various EOs or parent microemulsions. A similar capacity to remove the carbonaceous dirt is shown by the micellar systems using essential oils.

Although the optical images seem to indicate a relatively spotless appearance of the cleaned copper plates, the SEM images indicate the presence of residual deposits in some regions, suggesting that the proposed cleaning method, with a five-time repetition of wiping with a cotton swab, is not sufficient for complete removal of the dirt. Further studies must be performed to optimize the cleaning procedure, tailored to better fit with the characteristics of the artifact.

The SEM images recorded for Cu pieces covered with oily artificial dirt (MSC) show a more homogeneous layer deposited (Figure 4b) due to the lack of particulate matter in the composition and to the ability of oil and wax components to better adhere to the metallic surfaces. In most of the cases pertaining to the surface of the cleaned regions, the organic layer is absent or with minimal residues, suggesting a higher efficiency of P84-TPGS systems in the removal of greasy dirt.

The EDX analysis could also offer some semiquantitative information on the ability of a certain nanosystem to remove either grime artificial dirt or oily artificial dirt. Based on the EDX diagrams (Figure S2a,b), a reduction in the content of carbon found in the composition of the surface layer was computed as a cleaning efficiency indicator. In the case of the cleaning procedure performed on copper coupons affected by grime-like artificial dirt (MSA), a significant reduction in carbon percentage in the surface layer was observed compared to the composition before cleaning. The microemulsion with thyme oil lead to a reduction of 26.3%, while the one with cinnamon oil produced a similar decrease, of 21.7%. The EDX spectra recorded after the cleaning still showed a high percentage of C due to the presence of residual MSA artificial dirt, as it is observable in SEM images, and, additionally, due to the carbon content of the microemulsions organic components.

The EDX results obtained on the metallic plates coated with oily artificial dirt (MSC) showed a better capacity to decrease the carbon percentage in the interfacial layer. A reduction of 48.8% when cleaning with the P3 sample was recorded, similar to the value for sample P4 (52.9%). It was expected that the microemulsions containing xylene, with or without the addition of EOs, would exhibit an increased removal efficiency of oil and wax-based artificial dirt compared to the case of a more complex carbon black containing dirt. The results confirm the data in the SEM images, where MSC artificial dirt seems to be absent in the majority of images from the samples cleaned with microemulsions.

Quantitative assessment of cleaning cultural heritage items is currently performed based on color analysis using the variation of colorimetric parameters in the CIElab color space [42]. The chromatic coordinate L* describes brightness (from 0 for black to 100 for white), a* describes the red–green component (positive values for red and negative for green), while b* describes the yellow–blue component (positive for yellow and negative for blue). The total color variation is computed with Equation (1):

(1) $$\Delta E^{*}=\sqrt{\Delta L^{*2}+\Delta a^{*2}+\Delta b^{*2}}.$$

The variation of the chromatic coordinates was determined from the spectra registered on the surface of the copper plate coated with the artificial dirt versus the cleaned region of the same plate as the mean value of analysis of the collected spectra.

Similar color analysis was performed for the evaluation of the black soot removal from wall paintings located in the temple of Seti I (Abydos, Egypt), where the L* coordinate was considered the most relevant parameter to evidence the transition from the black spots to the original white color of the walls [43]. However, in the case of our experiments, due to the particular red color of the original copper coupons, the total chromatic variation ΔE* and the variation of the a* component are considered most suitable as chromatic characteristics to be analyzed. The higher the modification of the chromatic parameters, the more efficient of the cleaning is considered.

**Figure 5:**
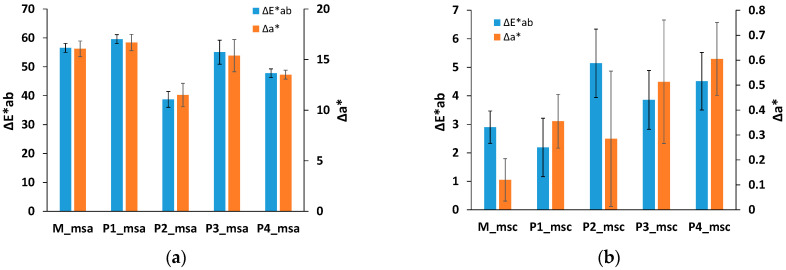
The variation of the chromatic coordinate Δa* and total color modification ΔE* for Cu coupons treated with grime artificial dirt (**a**) and oily artificial dirt (**b**) after cleaning with various nanosystems.

The variation of chromatic coordinates L*, a* and b* for copper plates in the region coated with artificial dirt compared to the regions subjected to the cleaning procedure is tabulated in Table S1. In Figure 5a,b, the influence of the composition of the cleaning agents on the values of total chromatic variation ΔE* and the variation of the red–green parameter Δa* after the cleaning procedure is shown.

The total color modification ΔE* values determined for the samples coated with grime-like artificial dirt and cleaned with a microemulsion (samples M, P3 and P4) are very close, with no statistically significant differences (*p* > 0.05), ranging from 56.55 ± 1.55 for xylene microemulsion to 55.06 ± 4.15 for thyme oil containing the microemulsion (sample P3) and 47.76 ± 1.53 for cinnamon oil containing the microemulsion (sample P4). A large value of the total color variation was also measured for the sample cleaned with mixed micelles loaded with thyme oil (sample P1), while in the case of the micellar system containing cinnamon oil a significant reduction in ΔE*, up to 38.72 ± 2.74, was observed. Possible explanations are the differences in the essential oil composition as chemical species exhibit various capacity to interact with the complex mixture of carbon black, oil, wax and natural polymer in the artificial dirt or the more compact layer of dirt that covered the copper coupon subjected to the cleaning procedure with sample P2.

The variation of total color ΔE* is due, in major part, to the variation of lightness parameter ΔL*, with high values, ranging from 41.68 to 53.12 for metallic plates coated with various P84-TPGS-based nanosystems with various EOs. The same abnormal behavior was recorded for cleaning with the P2 sample. The modification of lightness of the cleaned samples when using EOs containing microemulsions is similar to the effect of parent microemulsion with only xylene (*p* > 0.05), suggesting the same efficiency in the removal of carbon black dirt.

Regarding the red–green component analysis, a rather high value for a* parameter variation after cleaning was recorded for all systems, ranging from 13.51 ± 0.45 to 16.69 ± 0.81, without statistically significant difference (*p* > 0.05) for the presence of different oils. Again, a smaller value was obtained for the micelles encapsulating cinnamon essential oil.

These results suggest that the xylene microemulsion proved to be an efficient cleaning agent to remove the artificial dirt containing carbon black, and the addition of small amounts of an essential oil (either thyme or cinnamon oil) does not significantly change the cleaning results.

The values of variation of total color ΔE* produced by the cleaning procedure performed on oily artificial dirt were not significantly different from those observed when the xylene microemulsion or EO-loaded microemulsions were used. Similar values were obtained for micellar systems with EOs. In the same way, the variation of red–green component Δa* showed no significant differences between samples cleaned with various nanostructured systems containing various oils. The color analysis performed on the copper samples coated with oily artificial dirt and subjected to the cleaning procedure suggest that all P84-TPGS systems are able to remove greasy dirt, regardless of the nature of the oil encapsulated (xylene, EOs or both). Indeed, the combination of surfactants and synthetic or natural oils is an efficient way to solubilize organic nonpolar components of the artificial dirt and effectively remove them from surfaces due to a synergistic interaction between solubilization and detergency.

The deposition of artificial dirt on the copper surface lead to the change in surface hydrophobicity, which can be evaluated through contact angle variation. The variation of the hydrophobicity of the surface after the cleaning of the object is another method to evaluate the effectiveness of the removal of the deposited dirt.

**Figure 6:**
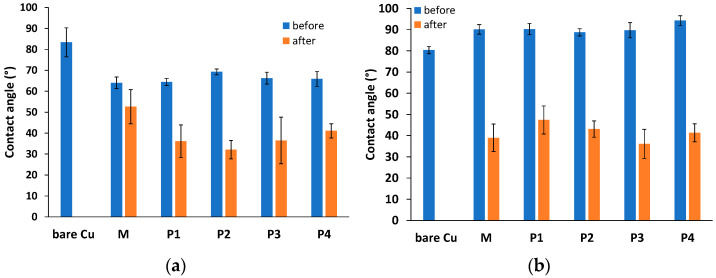
Water contact angles on Cu plates before and after cleaning procedure: (**a**) Cu coated with MSA (grime-like) artificial dirt and cleaned with various nanosystems; (**b**) Cu coated with MSC (oily) artificial dirt and cleaned with various nanosystems.

In Figure 6a,b, the static contact angle values are presented, for the copper coupons with grime-like artificial dirt and oily dirt before and after cleaning with various nanostructured systems containing P84-TPGS and various oils.

On a bare Cu plate, the average contact angle value for water was found to be 83.36 ± 6.89°, in the range of other data reported for copper samples with various roughness values and degrees of purity [44]. The deposited MSA artificial dirt lead to a significant decrease (*p* < 0.05) in the contact angle up to a main value of 65.98 ± 2.06°. The surface became more hydrophilic due to the characteristic of the carbon black particles in the artificial dirt composition. The removal of the MSA dirt with various microemulsion systems containing xylene or xylene and EOs (samples M, P3 and P4) lead to further decrease in the contact angle values, ranging from 32.10° to 52.62°. The decrease in the contact angle value was due to the replacement of the carbon-black-based deposited layer with surfactants and essential oils from the cleaning systems.

Pluronic derivatives were reported in the literature to produce a significant increase in the hydrophilicity when adsorbed on various surfaces [45]; thus, modification with Pluronics is a routine way to functionalize the polysulfone ultrafiltration membranes [46]. The mechanism involves a specific adsorption of the polymeric chains with the formation of brush-like features with polyoxyethylene fragments on the top. Vashita et al. [47] reports a decrease in the water contact angle on the electrospun PLGA nano-fibers from approximately 120° to approximately 20° when Pluronic P108 is blended with the PLGA. The same modification of the wettability of the surface is presumed to be produced by the other polymer in the composition of the microemulsion, namely TPGS, due to the presence of the hydrophilic residue of polyethylene glycol (PEG) in the molecule.

After cleaning with microemulsions containing both xylene and essential oils (P3 and P4), the contact angle values decreased to lower values compared to those caused by the effect of treatment with a microemulsion containing only xylene. This difference in hydrophilicity can be attributed to the distribution at the surface of various molecules from the essential oils.

As expected, the deposition of the MSC artificial dirt (oily dirt) increased the contact angle of the treated copper plates at an average value of 90.11 ± 5.04°. The CA value obtained for the copper coupon cleaned with pure microemulsion (sample M) reached 39.0 ± 6.44°, while in the case of the cleaning procedure performed with EO encapsulating microemulsions (samples P3 and P4), similar values were found (36.12 ± 6.89° for thyme-oil-loaded microemulsion and 41.37 ± 4.22° for cinnamon-oil-loaded microemulsion, respectively). Cleaning with micellar systems containing EOs has the same effect of increasing the hydrophilicity of the surface, with contact angles of 47.40 ± 6.62° for micelles with thyme oil and 43.09 ± 3.82° for micelles with cinnamon oil.

The decrease in the contact angles proves the removal of the greasy artificial dirt from the metal surfaces, followed by the adsorption of a thin layer of P84 and TPGS polymers, pure or blended with a small amount of phytochemicals from the essential oils.

### 3.5. Assessement of Anticorrosion Properties

Due to the numerous issues raised by the corrosion of metal historical objects and monuments, numerous techniques for preserving these artifacts were developed, the most common of which is the use of corrosion inhibitors. However, the majority of organic corrosion inhibitors are hazardous for human health, expensive and not eco-friendly. In recent years, research on green alternatives as corrosion inhibitors has advanced, reporting remarkable results with plant extracts. Among them, essential oils prove to be highly effective. Corrosion inhibitors usually act through a surface coating layer deposited on the metal surface. The mechanism involved in the anticorrosion effect of essential oils on Cu is based on the presence of electron-donating functional groups bearing N or O, together with the presence of aromatic groups, which are able to interact with the surface [48]. Thus, the addition of two EOs with a reported anticorrosion effect on a large variety of metals, i.e., thyme and cinnamon essential oils [49,50], in the obtained microemulsion was investigated.

Incralac laquer was used as a reference anticorrosion protective treatment, since it is one of the most frequently used technical products for metal conservation. In the composition of Incralac benzotriazole is the active anticorrosive ingredient, which is known to possess high toxicity and environmental risk.

Linear sweep voltammetry (LSV) was used to evaluate the corrosion process by sweeping the potential applied at a working electrode (Cu small coupons without and with several type of coatings) and measuring the current response. Electrochemical corrosion parameters were obtained and used for evaluation of the susceptibility of metallic support based on copper to corrosion.

**Figure 7:**
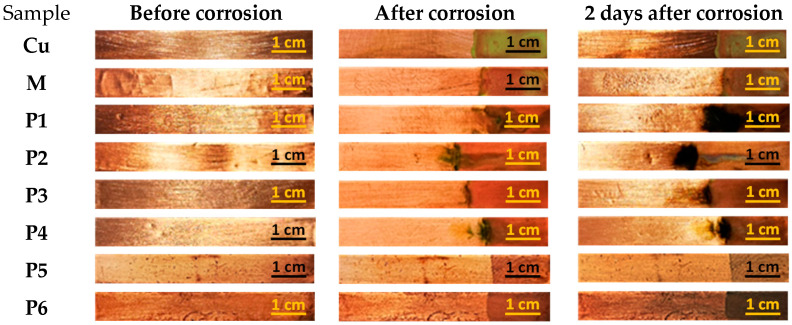
Copper samples coated with various P84-TPGS nanosystems during the corrosion test. Sample compositions of M, P1–P4 are described in Table 2. P5 and P6 are essential oils from thyme (P5) and from cinnamon (P6) used as model natural inhibitors of corrosion.

In Figure 7, the visual aspect of the copper samples subjected to the corrosion test is shown.

As can be observed in Figure 7, the green deposit specific for copper corrosion products is present on the bare Cu coupon, and, incidentally, on the one coated with xylene microemulsion (sample M), while on the metallic coupons coated with micelles containing EOs (samples P1 and P2) and the ones coated with microemulsions containing EOs (samples P3 and P4), the corrosion products are located in only several spots. As expected, no clear signs of the presence of corrosion products can be observed on the metallic coupons treated with undiluted essential oils (reference samples P5 and P6).

**Figure 8:**
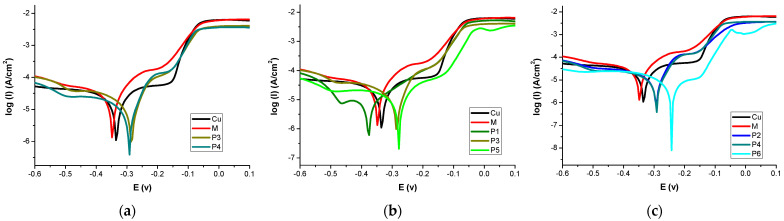
Potentiodynamic polarization curves for copper coupons coated with various P84-TPGS nanosystems loaded with essential oils. (**a**) Polarization curves for Cu coupons coated with Xylene microemulsion (M) and Xy microemulsion containing EOs (thyme oil sample P1, cinnamon oil sample P2); (**b**) Polarization curves for Cu coupons coated with thyme oil containing various systems (surfactant micelles encapsulating thyme oil P1, microemulsion containing thyme oil P3 and pure thyme oil P5); (**c**) Polarization curves for Cu coupons coated with cinnamon oil containing various systems (surfactant micelles encapsulating cinnamon oil P2, microemulsion containing cinnamon oil P4 and pure cinnamon oil P6).

The Tafel polarization curves of the uncoated and coated copper plates were obtained after their exposure to the sodium chloride solution (NaCl 3.5%wt). The results are presented in Figure 8 for the different types of protective coatings.

The corrosion parameters including the polarization resistance (Rp), corrosion potential (E_corr_), and corrosion current density (I_corr_) are collected in Table S2 from Supplementary Information.

Data from Table S2 show that essential oils extracted from thyme and cinnamon provide good protection for copper in a 3.5%wt NaCl solution, and this behavior is preserved when EOs are encapsulated in either P84-TPGS micelles or in microemulsion.

From Figure 8b,c, it can be observed that the corrosion potentials shifted to more positive values for Cu coupons coated with pure EOs (samples P5 and P6) and EOs in micelles or microemulsions (P2–P4). One exception waas the shift to a more negative value for the Cu treated with thyme oil loaded in mixed micelles (sample P1, accordingly). An inhibitor can be classified as cathodic or anodic if the difference between corrosion potentials (E_corr_) is above 85 mV [51].

We note that the E_corr_ difference between P6 and the blank sample (Cu) was equal to 91 mV, indicating that cinnamon oil acts as an anodic-type inhibitor. For other samples, the difference between corrosion potentials was smaller than 85 mV, indicating a mixed-type inhibitor.

The inhibitor molecules from EOs can be adsorbed on the copper surface in the form of species with negative charge which can interact by electrostatic forces with a positively charged metal surface. The surface coverage was increased and consequently protected against corrosion.

The effectiveness of inhibition (EI%) could be calculated from current densities I_corr_ using Equation (2):

(2) $$EI\left(\%\right)=\frac{I_{corr}-I_{corr}^{\prime }}{I_{corr}}\times 100,$$

where I_corr_ and I′_corr_ represent, respectively, the current corrosion densities at the corrosion potential without and with the different type of coatings. These values were determined by extrapolation using the Tafel method with a potential range of 100 mV around the corrosion potential.

As expected, the highest values for the effectiveness of corrosion inhibition were obtained for treatments made from pure essential oils, 96.2% for thyme oil and 99.0% for cinnamon oil, respectively.

It is to be noted that the microemulsion containing only xylene as the oily phase, without EOs, itself exhibited a rather good anticorrosion effect, with an effectiveness of inhibition value of 59.3. The increased values of EI were maintained for both essential oils encapsulated in micelles (90.6% and 92.1% for samples with thyme EO and with cinnamon EO, respectively). The same behavior was observed in the case of the microemulsion containing cinnamon EO, with a value of effectiveness of inhibition of 94.5%, while a small decrease was registered for the treatment with the microemulsion loaded with thyme oil (EI of 82.0%). For all samples, we observed a decrease in current densities and corrosion rates, the effectiveness of inhibition being greater than 90% for almost all films containing essential oils. This demonstrated the remarkable anticorrosive protection, higher than the 85% effectiveness of inhibition produced by the application of the Incralac commercial product. Best results were obtained for copper covered with cinnamon oil (>99%); this oil is an effective anodic corrosion inhibitor of copper in a solution of 3.5%wt NaCl [52].

Theoretical studies showed that some components such as carvacrol, methyl ether and borneol extracted from thyme can be also very good inhibitors of metal corrosion [53].

**Figure 9:**
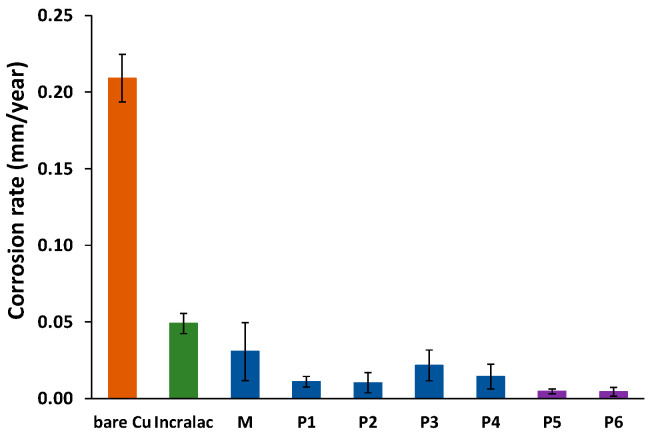
Corrosion rate of copper coupons treated with cleaning nanosystems containing EOs.

The variation of the corrosion rate estimated from Tafel curves for Cu coupons treated with different nanosystems encapsulating EOs is shown in Figure 9, compared to the effect of the pure essential oils.

Compared to bare copper, the metallic coupons coated with essential oils, either in pure form or embedded in micelles or in microemulsions, exhibited statistically significant lower corrosion rates (*p* < 0.05). It is to be mentioned that the xylene microemulsion (M) itself was able to reduce the copper corrosion rate from 0.209 ± 0.016 mm/year to 0.031 ± 0.019 mm/year, to a greater extent compared with the effect of Incralac anticorrosive coating, which lead to a corrosion rate of 0.049 ± 0.007 mm/year. The application of pure EOs on the copper surfaces lead to a spectacular decrease in the corrosion rate of the metallic coupons, up to 0.0047 ± 0.0015 and 0.0043 ± 0.0029 mm/year for thyme EO and for cinnamon EO, respectively. Encapsulation of a small amount of the essential oils in micelles or microemulsions produced a decrease in the anticorrosion effect, as was expected. However, the decrease on the corrosion rate is still very important, for all samples the values are in the range of 0.0103 to 0.0216, with no significant differences due to nanocarriers.

The results of the electrochemical study revealed that thyme and cinnamon essential oils encapsulated in mixed P84-TPGS micelles or in the obtained P84-TPGS-based microemulsions are able to provide anticorrosion protection when applied to copper model surfaces, which is very similar to the effect of the pure EOs.

### 3.6. Antifungal Activity

Historic and artistic objects deposited in unsuitable conditions or exposed to outdoor conditions are often subjected to microorganism colonization. In particular, *A. niger* is one of the most aggressive fungal species that cause biodeterioration of copper cultural heritage items.

Antifungal activity of the thyme- and cinnamon-EO-containing nanosystems (P84-TPGS mixed micelles and P84-TPGS-based microemulsion) was evaluated in order to emphasize the potential of the proposed cleaning agents to provide an additional benefit for the protection of copper artifacts.

Minimum inhibitory concentrations of EO-loaded nanosystem were determined and the results are shown in Figure S3 in Supplementary Materials. Microemulsion containing xylene and mixed micelles from P84-TPGS mixtures showed no significant antifungal activity against *A. niger*, with a determined value of MIC > 100 mg/mL. The samples containing essential oils (mixed micelles loaded with thyme oil and with cinnamon oil, P1 and P2 and microemulsions with the mentioned EOs, P3 and P4, respectively) showed a moderate antifungal activity, with MIC values in the range of 13.3 mg/mL to 26.8 mg/mL. These modest results are consistent with the very low content of EOs in the nanosystems (approximately 1%). The similar antifungal effect of EOs encapsulated in micelles and in microemulsions suggests that the carrier’s composition did not affect the biological efficiency of the encapsulated fitochemicals.

**Figure 10:**
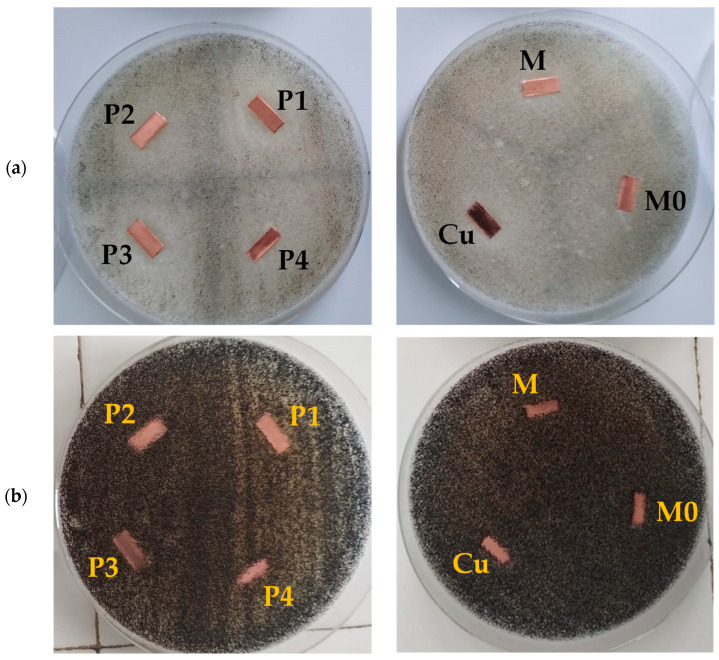
The macroscopic aspect of *Aspegillus niger* strain culture in contact with treated copper plates: (**a**) *A. niger* culture after 3 days of incubation; (**b**) *A. niger* culture after 7 days of incubation. M and M0 are microemulsion with xylene and pure P84-TPGS micelles used as references. Composition of samples P1–P4 is detailed in Table 2.

Figure 10 shows the optical images of the *A. niger* cultures incubated with the nanosystems containing EOs at concentration equal to MIC and a lower one (minimal subinhibitory concentration MSIC).

The growth of the fungi was evidenced in the samples exposed to a microemulsion with xylene and to void micelles, with similar results to those of the *A. niger* control sample.

The samples treated with EOs in both mixed micelles and microemulsions produced a significant inhibition of the fungal hyphae development, with no significant differences between essential oils or nanosystems.

In Figure 10, qualitative results of the fungi growth inhibition of the various nanosystems containing EOs deposited on the copper plates are presented.

The bare Cu plate and the coupons treated with nanosystems without EOs show evidence of fungal colonization (Figure 10a) from the first 3 days of incubation, while the metallic plates treated with either micelles or microemulsions containing EOs are less affected by the microbial growth. The unprotected (bare) Cu coupon is entirely covered by fungal culture, while the plates treated with surfactant micelles or with microemulsion exhibit a certain protective effect, probably due to the oil and surfactant deposited layer that acts as a barrier against humidity, and slows microorganism growth. After 7 days of incubation, the copper samples treated with microemulsions and micelles encapsulating EOs still show little signs of colonization, with the exception of sample P4, a microemulsion containing cinnamon oil, which seems to lose in a certain degree the fungicidal efficiency.

**Figure 11:**
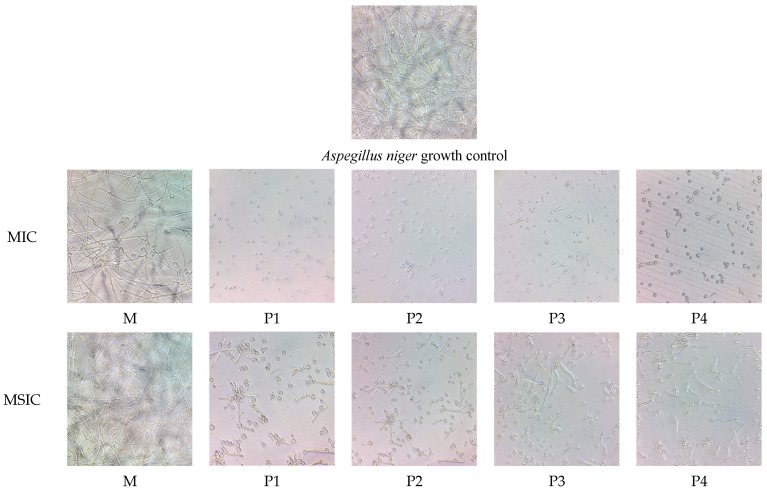
Optical microscope micrographs (magnification 100×) of *A. niger* filaments in cultures incubated with nanosystems at MIC (**top**) and with minimal subinhibitory concentration (MSIC) (**bottom**).

The inhibition of microorganism growth can also be confirmed microscopically, by evidencing the fungal filaments aspect (Figure 11). The position of the samples subjected to the microscopic evaluation on the culture plates is presented in Figure S4.

The essential oils used in the present study show a composition (as declared by the producer) similar to the ones reported in the literature, i.e., the main components of thyme EO are thymol, *p*-cymene, *p*-mentha-1,4-diene and carvacrol, while the cinammon EO contains eugenol benzyl benzoate and cinnamaldehyde. Generally, the composition of the essential oils is very complex, including chemical compounds from different classes such as terpenoids, aldehydes and ketones, esters and alcohols in various proportions. Thus, it is very difficult to clearly assign the antimicrobial activity to a certain component. However, biological properties could be attributed to major components; in particular, for thyme and cinnamon EOs, they could be attricuted to thymol, carvacrol and eugenol.

The common conclusion in the literature is that the synergistic effect due to the complex composition explains the spectacular antifungal and antibacterial properties of essential oils; thus, for practical use, the entire natural product (essential oil) is recommended instead of pure components.

Despite the increasingly serious concern regarding the severe effects of *A. niger* that produce biodeterioration on cultural heritage objects and monuments [20,54], research is still focused on the analysis of the effects of microbial colonization, and far fewer studies are dedicated to the destruction or prevention of fungal attacks [55].

Along with synthetic fungicides such as azole derivatives, some essential oils have also been tested in recent years for preventive antifungal or fungicidal treatment of artistic objects or documents, but the respective materials were wood, paper or stone [56,57]. According to our knowledge, no research has been published regarding the protection of metal cultural heritage objects with essential oils, so we do not have the possibility to compare our results.

In the experimental conditions of the present study, i.e., the cleaning procedure applied to copper model plates using various nanosystems containing very low amounts of essential oils, the layer deposited on the metallic surfaces as a residual cleaning agent is enough to ensure good protection against colonization with *A. niger*. A better effect could be obtained if, at the end of cleaning procedure, a rinsing additional step were to be performed with pure essential oil.

## 4. Conclusions

In this research, novel microemulsions with essential oils are proposed as environmentally friendly cleaning solutions for metallic historic objects. For the first time, the systems containing a combination of water, ethanol, xylene, Pluronic P84 and TPGS as a surfactant mixture were investigated for the ability to obtain single-phase microemulsions. The phase diagram shows the existence of an O/W single-phase microemulsion in the region with low xylene and surfactant content, while further increase in P84-TPGS concentration leads to the formation of highly viscous phases, probably lyotropic liquid crystals. A microemulsion with minimum surfactant and oil concentrations (11.4% and 4.5%) was selected for further studies, with or without encapsulated essential oils. EO loading microemulsions were successfully prepared, encapsulating up to 1.1% thyme or cinnamon oils.

The cleaning procedure, performed with cotton swabs soaked in microemulsions on the model copper coupons covered with artificial dirt, leads to the efficient removal of two types of dirt. The cleaning capacity evaluated from color analysis and variation of wettability is similar for parent microemulsions compared to those also encapsulating EOs. All three microemulsions have better ability to clean the oily artificial dirt, while the black carbon containing artificial dirt is more difficult to entirely remove. Further studies are needed in order to optimize the cleaning procedure by comparing more methods currently in use in conservation of the metal artistic works of art. The contact angle measurement on the Cu plates before and after cleaning suggests that artificial dirt was removed from the surface, and a thin layer of organic component from the microemulsion was formed, which is considered advantageous for protection of historic objects until the final stages of restoration.

The addition of essential oils from thyme or cinnamon leaves turns the parent microemulsion into a multifunctional tool with anticorrosion and antimicrobial properties.

Even at reduced percentages of EOs, the microemulsions deposited on the copper coupons exhibit significant protection against corrosion, as it is shown by the results of the electrochemical measurements. As expected, the determined effectiveness of corrosion inhibition by EO-loaded microemulsions is lower than the effect produced by pure essential oils. However, microemulsions with encapsulated EOs exhibit remarkable anticorrosion protective efficiency due to the intrinsic effect of parent microemulsion. It was not possible to compare our data with that of the literature, since no similar studies on the anti-corrosion protection of coatings based on microemulsions with volatile oils were found.

The prepared P84-TPGS microemulsion exhibits no biological activity against the tested fungal strain (*A. niger*), while the encapsulation of 1% EOs in the parent microemulsion improves its ability to inhibit microbial growth on treated metallic plates.

In conclusion, for the first time, microemulsion systems from P84 and TPGS, with and without essential oils, are investigated as potential tools in the cleaning of metallic historic objects.

The novel microemulsion formulated with a blend of nontoxic surfactants allows a significant reduction in the harmful organic solvent in a cleaning agent suitable for use in cultural heritage conservation, minimizing the health risks of the exposed practitioners. The EO encapsulating microemulsions proposed combine cleaning efficiency for both greasy and grime-like artificial dirt with consistent protective properties against corrosion and fungal attacks. Further research is required to optimize the strategy of cleaning with such microemulsions in terms of tailoring the composition based on a particular type of dirt contaminating the artifacts and the frequency of cleaning sequences.

## Figures and Tables

**Table 1:** Chemical formula and characteristics of used surfactant.

Surfactant	Chemical Formula	MW	HLB	CMC ^1^
Pluronic P84	(PEO)_19_–(PPO)_43_–(PEO)_19_	4200	14	1.9 × 10^−4^ M
TPGS	(C_2_H_4_O)_n_–C_33_H_54_O_5_	1513	13	1.0 × 10^−6^ M

^1^ The CMC values are experimentally determined and reported in a previous study [33].

**Table 2:** Composition of the nanosystems selected for cleaning efficiency evaluation.

Sample Code	Concentration % (*w*/*w*)				
	Water/EtOH	S_mix_	Xylene	Thyme EO	Cinnamon EO
M	84.11	11.35	4.54	0	0
P1	87.25	11.78	0	0.97	0
P2	87.13	11.76	0	0	1.11
P3	83.32	11.25	4.50	0.93	0
P4	83.21	11.23	4.49	0	1.06

**Table 3:** Size, size distribution and zeta potential of the nanosystems based on P84-TPGS surfactant mixtures with xylene and essential oils (values are mean ± SD, *n* = 3). Sample composition in Table 2.

Sample Code	Size (nm)	PdI	Zeta Potential (mV)
M	19.16 ± 0.88	0.40 ± 0.01	−1.18 ± 0.03
P1	12.65 ± 1.09	0.77 ± 0.05	−1.10 ± 0.14
P2	11.24 ± 1.75	0.71 ± 0.03	−2.19 ± 0.46
P3	23.08 ± 0.5	0.48 ± 0.02	−0.74 ± 0.07
P4	42.89 ± 2.59	0.49 ± 0.03	−0.96 ± 0.11

## Data Availability

Not Applicable.

## Supplementary Materials

The following supporting information can be downloaded at: https://www.mdpi.com/article/10.3390/nano13172430/s1, Figure S1: Size and size distribution in number for the xylene microemulsion (sample M – red line) and xylene microemulsion with encapsulated thyme essential oil (sample P3 – green line); Figure S2: EDX images of Cu coupons covered with grime-like artificial dirt (upper register) and oily artificial dirt (bottom register), before and after cleaning with encapsulating Thyme EO microemulsions; (a) and after cleaning with encapsulating cinnamon EO microemulsions (b); Figure S3: MIC values of various P84-TPGS nanosystems tested against *A. niger*; Figure S4. (a): Antifungal activity against *A. niger* of P84-TPGS micellar dispersion and of P84-TPGS microemulsion with xylene, (b): Antifungal activity against *A. niger* of P84-TPGS micellar dispersions with EOs (P1 with thyme, P2 with cinnamon), (c): Antifungal activity against *A. niger* of xylene P84-TPGS microemulsions with EOs (P3 with thyme, P4 with cinnamon); Table S1: The chromatic coordinated L*, a* and b* variation determined for copper coupons treated with grime artificial dirt (MSA) and oily artificial dirt (MSC) after cleaning with various P84-TPGS nanosystems (micelles and microemulsions).; Table S2: Corrosion parameters determined for Cu coupons coated with different P84-TPGS nanosystems.
