# Supplementary material for: Novel Microemulsions with Essential Oils for Environmentally Friendly Cleaning of Copper Cultural Heritage Artifacts
Source: Nanomaterials (Basel). 2023 Aug 26;13(17):2430. doi: 10.3390/nano13172430 (PMC10490116; doi:10.3390/nano13172430)
Supplement: Supplementary file 1 [file nanomaterials-13-02430-s001.zip › nanomaterials-2558932-supplementary.pdf]

Supplementary informations

Results

			Size (d.nm):	% Number:	St Dev (d.n...
Z-Average (d.nm):	67.54	Peak 1:	14.36	100.0	3.677
PdI:	0.459	Peak 2:	0.000	0.0	0.000
Intercept:	0.954	Peak 3:	0.000	0.0	0.000
Result quality :	Refer to quality report				

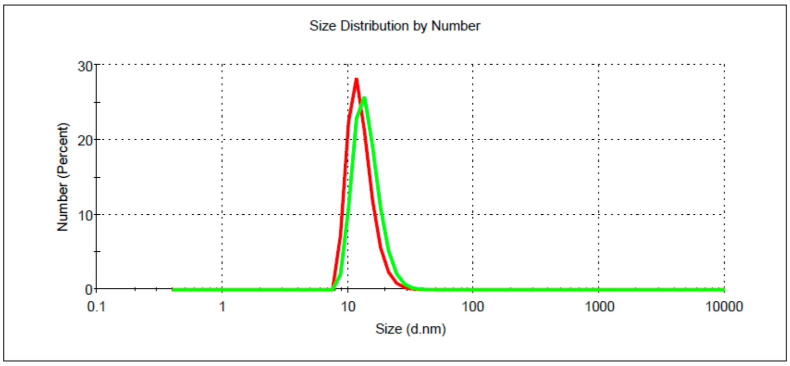

Figure S1. Size and size distribution in number for the xylene microemulsion (sample M – red line) and xylene microemulsion with encapsulated thyme essential oil (sample P3 – green line).

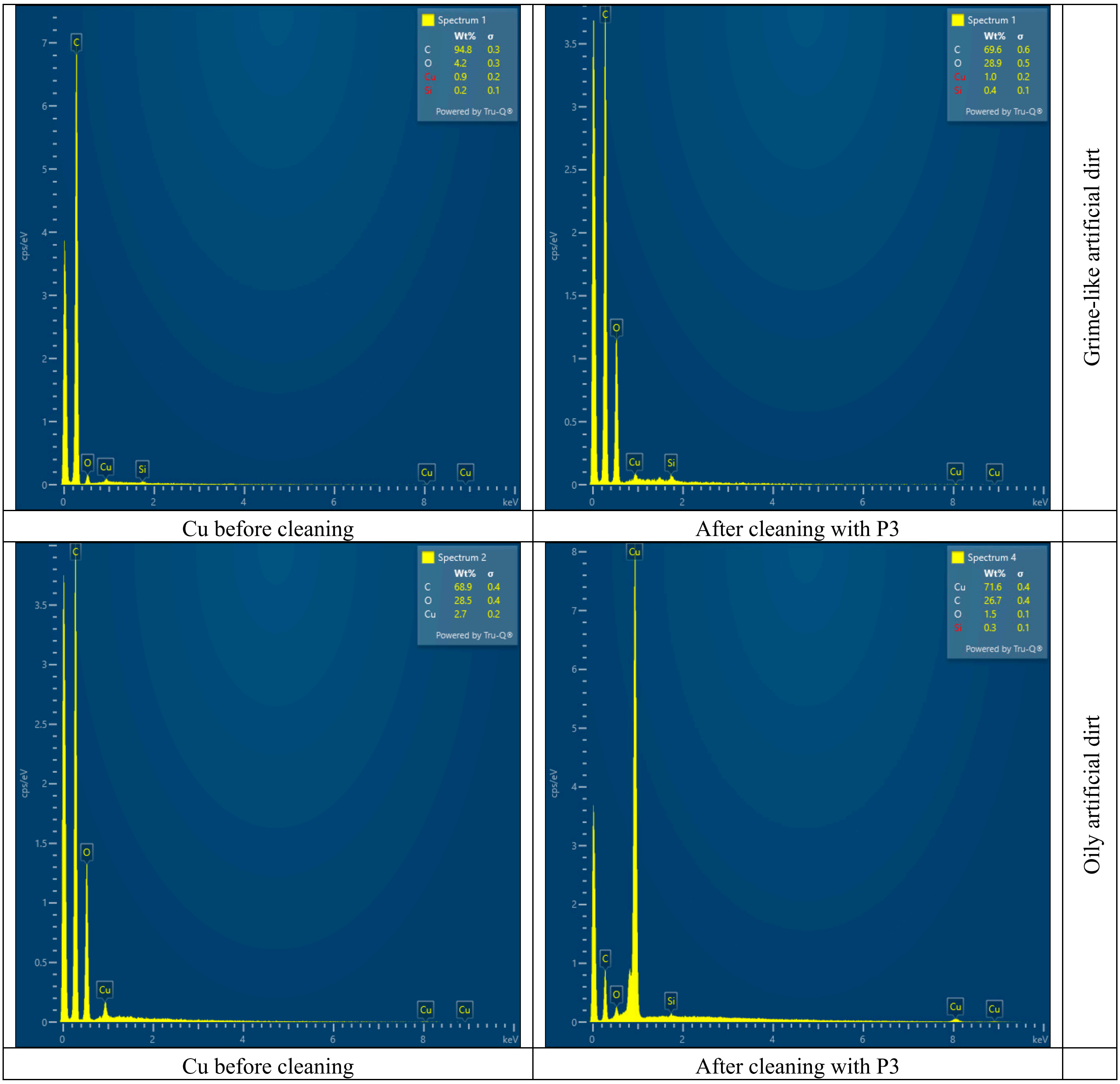
Spectrum 1
Wt% σ
C 94.8 0.3
O 4.2 0.3
Cu 0.9 0.2
Si 0.2 0.1
Powered by Tru-Q®
cps/eV
keV
Cu before cleaning
Spectrum 1
Wt% σ
C 69.6 0.6
O 28.9 0.5
Cu 1.0 0.2
Si 0.4 0.1
Powered by Tru-Q®
After cleaning with P3
Grime-like artificial dirt
Spectrum 2
Wt% σ
C 68.9 0.4
O 28.5 0.4
Cu 2.7 0.2
Powered by Tru-Q®
Cu before cleaning
Spectrum 4
Wt% σ
Cu 71.6 0.4
C 26.7 0.4
O 1.5 0.1
Si 0.3 0.1
Powered by Tru-Q®
After cleaning with P3
Oily artificial dirt

(a)

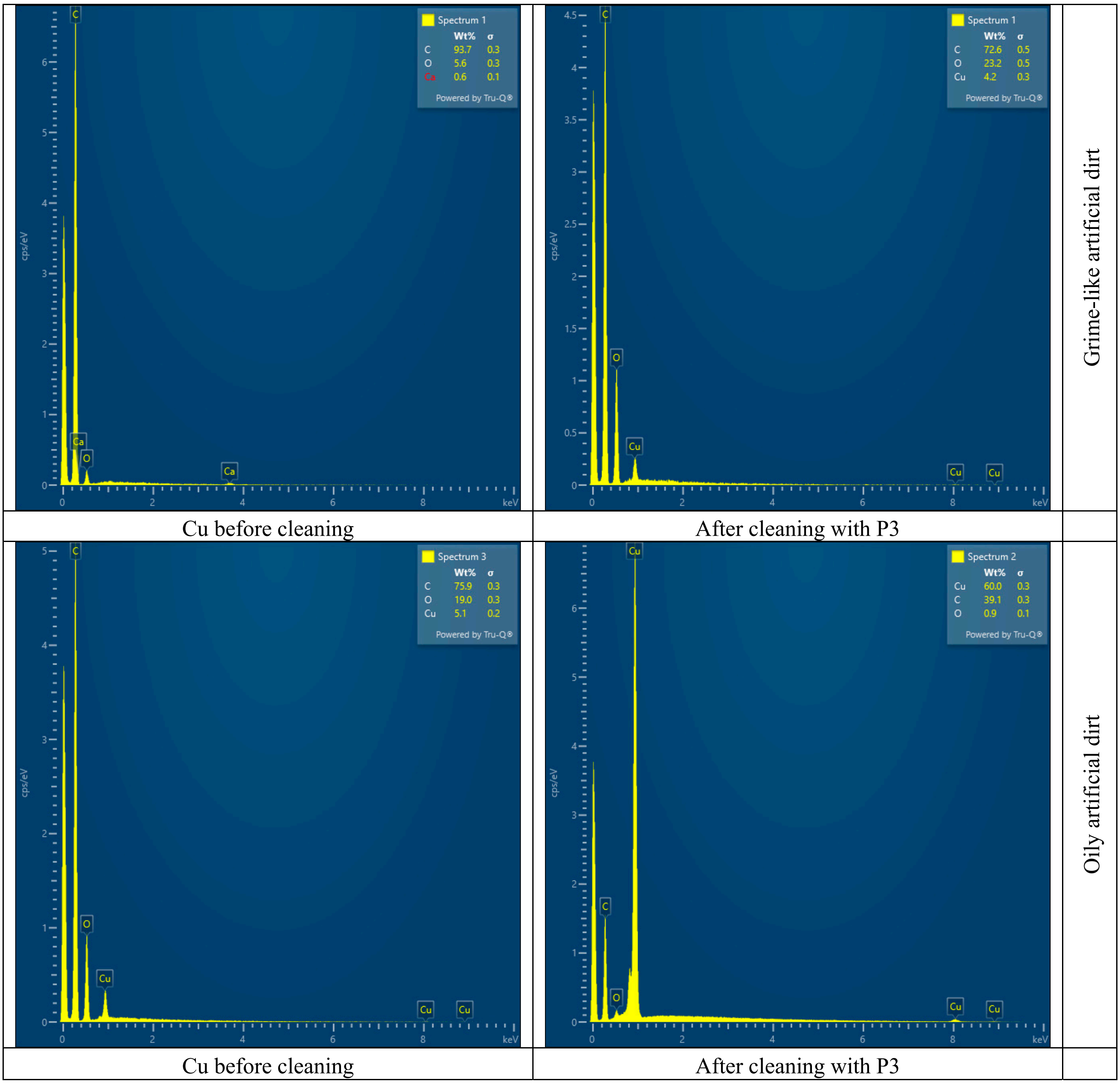

(**b**)

Figure S2. EDX images of Cu coupons covered with grime-like artificial dirt (upper register) and oily artificial dirt (bottom register), before and after cleaning with encapsulating Thyme EO microemulsions; (**a**) and after cleaning with encapsulating cinnamon EO microemulsions (**b**).

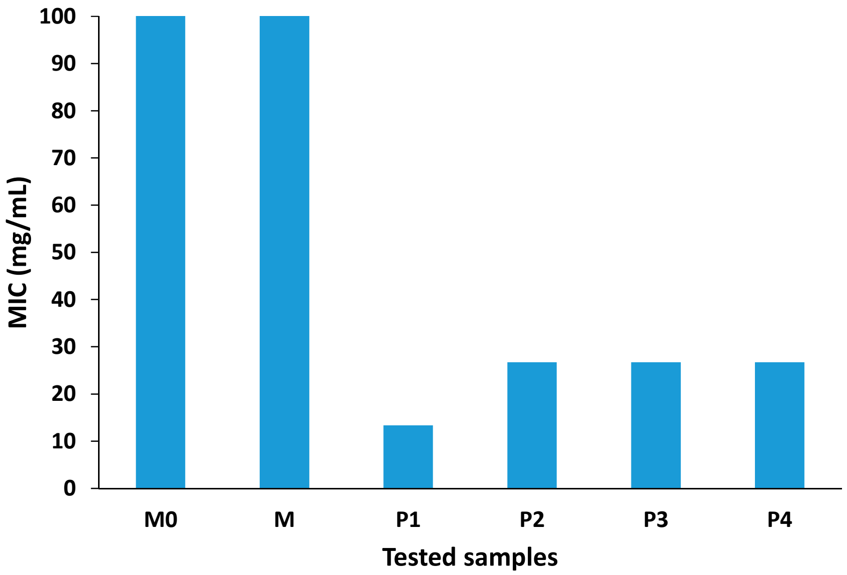

Figure S3. MIC values of various P84-TPGS nanosystems tested against *A. niger*.

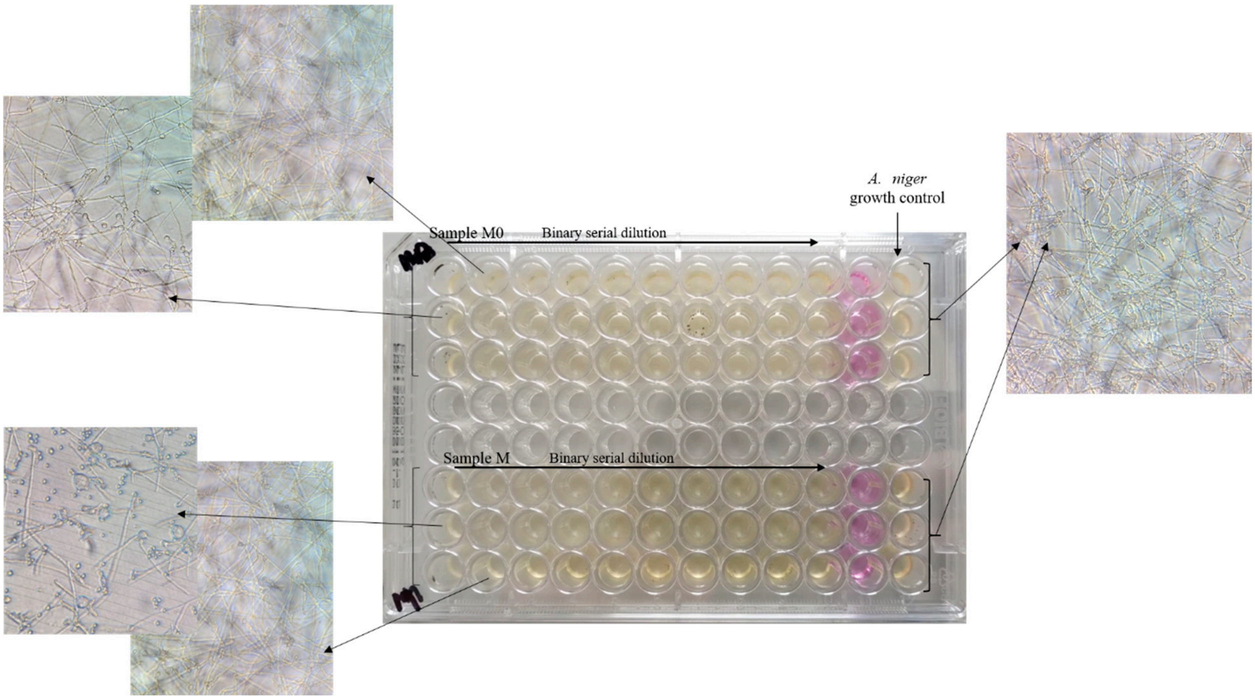
A. niger
growth control
Sample M0
Binary serial dilution
Sample M
Binary serial dilution

(**a**)

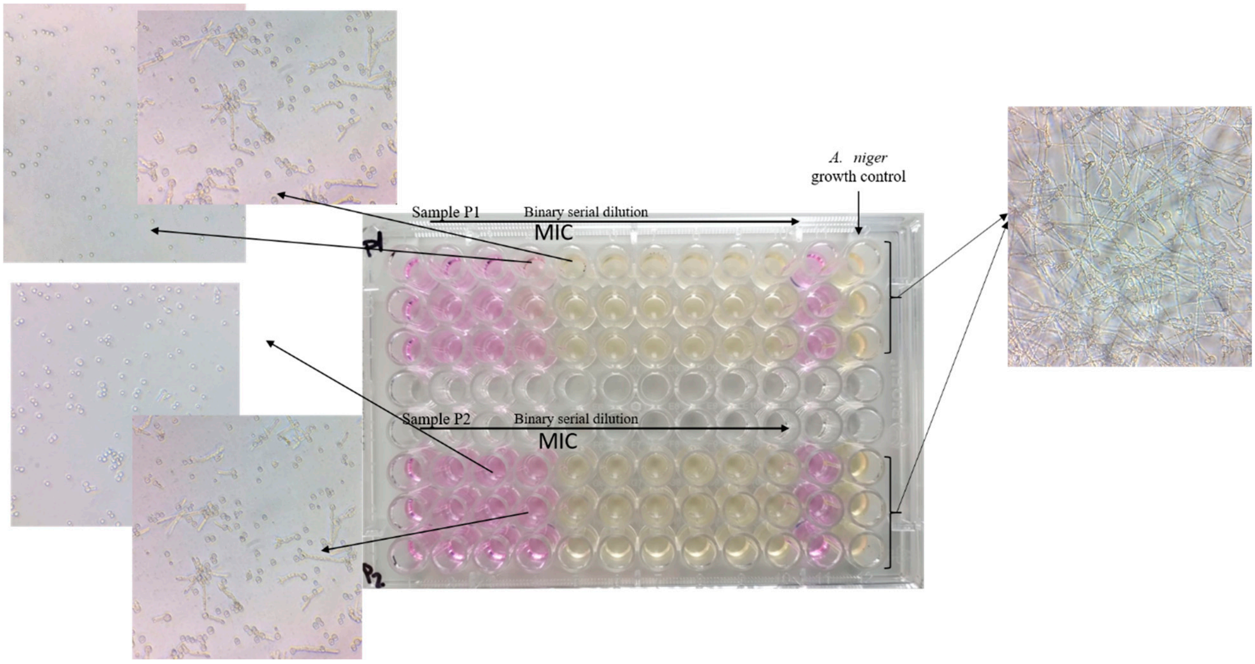
A. niger
growth control
Sample P1
Binary serial dilution
MIC
Sample P2
Binary serial dilution
MIC

(**b**)

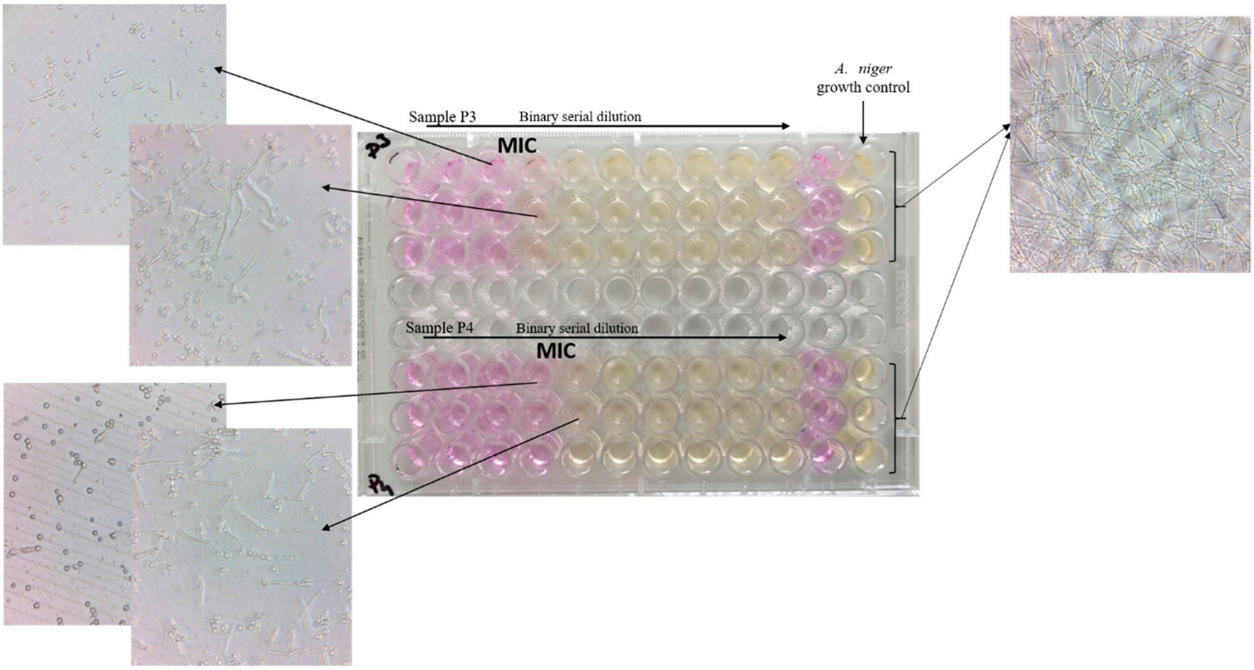

(**c**)

Figure S4. (**a**): Antifungal activity against *A. niger* of P84-TPGS micellar dispersion and of P84-TPGS microemulsion with xylene, (**b**): Antifungal activity against *A. niger* of P84-TPGS micellar dispersions with EOs (P1 with thyme, P2 with cinnamon), (**c**): Antifungal activity against *A. niger* of xylene P84-TPGS microemulsions with EOs (P3 with thyme, P4 with cinnamon).

Table S1. The chromatic coordinated L*, a* and b* variation determined for copper coupons treated with glime artificial dirt (MSA) and oily artificial dirt (MSC) after cleaning with various P84-TPGS nanosystems (micelles and microemulsions).

Cleaning agent	Artificial dirt	ΔL*	Δa*	Δb*
M	MSA	50.59 ± 1.08	16.06 ± 0.77	19.44 ± 1.01
P1	MSA	53.12 ± 1.08	16.69 ± 0.8	21.09 ± 1.01
P2	MSA	33.36 ± 2.1	11.49 ± 1.15	15.92 ± 1.42
P3	MSA	48.97 ± 3.37	15.38 ± 1.59	19.92 ± 1.95
P4	MSA	41.68 ± 1.35	13.50 ± 0.44	19.00 ± 0.54
M	MSC	0.31 ± 0.13	0.09 ± 0.08	1.22 ± 0.21
P1	MSC	-0.50 ± 0.58	0.35 ± 0.10	1.48 ± 0.23
P2	MSC	-2.02 ± 1.09	0.29 ± 0.27	4.60 ± 1.03
P3	MSC	-3.56 ± 3.14	0.51 ± 0.24	2.12 ± 1.78
P4	MSC	-1.70 ± 1.38	0.61 ± 0.14	3.96 ± 0.68

Table S2. Corrosion parameters determined for Cu coupons coated with different P84-TPGS nanosystems.

Sample	Composition of coating on Cu coupons	R_{ct} (Ω)	$-E_{corr}$ (V)	I_{corr} (μA/cm^2)
M	μE with xylene	107.40 ± 72.72	0.38 ± 0.12	2.99 ± 1.79
P1	P84-TPGS micelles with thyme EO	90.35 ± 28.07	0.31 ± 0.05	1.08 ± 0.33
P2	P84-TPGS micelles with cinnamon EO	98.14 ± 41.94	0.35 ± 0.12	1.04 ± 0.66
P3	μE with xylene and thyme EO	161.5 ± 36.91	0.36 ± 0.13	2.10 ± 1.01
P4	μE with xylene and cinnamon EO	52.79 ± 20.25	0.31 ± 0.03	1.40 ± 0.77
P5	Thyme essential oil	98.28 ± 37.24	0.28 ± 0.01	0.51 ± 0.15
P6	Cinnamon essential oil	80.87 ± 23.40	0.26 ± 0.02	0.47 ± 0.34
